# Alterations in T and B cell function persist in convalescent COVID-19 patients

**DOI:** 10.1016/j.medj.2021.03.013

**Published:** 2021-06-11

**Authors:** Halima A. Shuwa, Tovah N. Shaw, Sean B. Knight, Kelly Wemyss, Flora A. McClure, Laurence Pearmain, Ian Prise, Christopher Jagger, David J. Morgan, Saba Khan, Oliver Brand, Elizabeth R. Mann, Andrew Ustianowski, Nawar Diar Bakerly, Paul Dark, Christopher E. Brightling, Seema Brij, CIRCO, Rohan Ahmed, Miriam Avery, Katharine Birchall, Evelyn Charsley, Alistair Chenery, Christine Chew, Richard Clark, Emma Connolly, Karen Connolly, Simon Dawson, Laura Durrans, Hannah Durrington, Jasmine Egan, Kara Filbey, Claire Fox, Helen Francis, Miriam Franklin, Susannah Glasgow, Nicola Godfrey, Kathryn J. Gray, Seamus Grundy, Jacinta Guerin, Pamela Hackney, Chantelle Hayes, Emma Hardy, Jade Harris, Anu John, Bethany Jolly, Verena Kästele, Gina Kerry, Sylvia Lui, Lijing Lin, Alex G. Mathioudakis, Joanne Mitchell, Clare Moizer, Katrina Moore, Stuart Moss, Syed Murtuza Baker, Rob Oliver, Grace Padden, Christina Parkinson, Michael Phuycharoen, Ananya Saha, Barbora Salcman, Nicholas A. Scott, Seema Sharma, Jane Shaw, Joanne Shaw, Elizabeth Shepley, Lara Smith, Simon Stephan, Ruth Stephens, Gael Tavernier, Rhys Tudge, Louis Wareing, Roanna Warren, Thomas Williams, Lisa Willmore, Mehwish Younas, Timothy Felton, Angela Simpson, John R. Grainger, Tracy Hussell, Joanne E. Konkel, Madhvi Menon

**Affiliations:** 1 Lydia Becker Institute of Immunology and Inflammation, Division of Infection, Immunity, and Respiratory Medicine, School of Biological Sciences, Faculty of Biology, Medicine, and Health, University of Manchester, Manchester Academic Health Science Centre, Room 2.16, Core Technology Facility, 46 Grafton Street, Manchester, M13 9PL, UK; 2 Institute of Immunology and Infection Research, School of Biological Sciences, University of Edinburgh, Ashworth Laboratories, Edinburgh EH9 3FL, UK; 3 Department of Respiratory Medicine, Salford Royal NHS Foundation Trust, Stott Lane, Salford M6 8HD, UK; 4 Wellcome Trust Centre for Cell Matrix Research, The University of Manchester, Manchester M13 9PT, UK; 5 Division of Infection, Immunity, and Respiratory Medicine, Manchester NIHR BRC, Education and Research Centre, Wythenshawe Hospital, Manchester, UK; 6 Maternal and Fetal Health Centre, Division of Developmental Biology, School of Medical Sciences, Faculty of Biology, Medicine, and Health, The University of Manchester, 5th Floor, St. Mary’s Hospital, Oxford Road, Manchester M13 9WL, UK; 7 Regional Infectious Diseases Unit, North Manchester General Hospital, Manchester, UK; 8 Department of Respiratory Sciences, University of Leicester, Leicester LE3 9QP, UK; 9 Department of Respiratory Medicine, Manchester Royal Infirmary, Manchester University NHS Foundation Trust, Manchester, UK; 10 Intensive Care Department, Salford Royal NHS Foundation Trust, Stott Lane, Salford M6 8HD, UK

**Keywords:** COVID-19, T cells, B cells, long COVID, viral Infection, convalescent patients

## Abstract

**Background:**

Emerging studies indicate that some coronavirus disease 2019 (COVID-19) patients suffer from persistent symptoms, including breathlessness and chronic fatigue; however, the long-term immune response in these patients presently remains ill-defined.

**Methods:**

Here, we describe the phenotypic and functional characteristics of B and T cells in hospitalized COVID-19 patients during acute disease and at 3–6 months of convalescence.

**Findings:**

We report that the alterations in B cell subsets observed in acute COVID-19 patients were largely recovered in convalescent patients. In contrast, T cells from convalescent patients displayed continued alterations with persistence of a cytotoxic program evident in CD8^+^ T cells as well as elevated production of type 1 cytokines and interleukin-17 (IL-17). Interestingly, B cells from patients with acute COVID-19 displayed an IL-6/IL-10 cytokine imbalance in response to Toll-like receptor activation, skewed toward a pro-inflammatory phenotype. Whereas the frequency of IL-6^+^ B cells was restored in convalescent patients irrespective of clinical outcome, the recovery of IL-10^+^ B cells was associated with the resolution of lung pathology.

**Conclusions:**

Our data detail lymphocyte alterations in previously hospitalized COVID-19 patients up to 6 months following hospital discharge and identify 3 subgroups of convalescent patients based on distinct lymphocyte phenotypes, with 1 subgroup associated with poorer clinical outcome. We propose that alterations in B and T cell function following hospitalization with COVID-19 could affect longer-term immunity and contribute to some persistent symptoms observed in convalescent COVID-19 patients.

**Funding:**

Provided by UKRI, Lister Institute of Preventative Medicine, the Wellcome Trust, The Kennedy Trust for Rheumatology Research, and 3M Global Giving.

## Highlights

Lymphocytes were examined during COVID-19 and at up to 6 months of convalescence
B cell changes seen during acute COVID-19 were largely restored in convalescence
T cells from convalescent COVID-19 patients displayed persistent changes
Lymphocyte signatures defined 3 convalescent patient groups, one with poorer outcomes

## Context and significance

The coronavirus disease 2019 (COVID-19) pandemic, caused by a novel coronavirus strain, has resulted in >100 million infections worldwide. Emerging evidence suggests that some COVID-19 patients suffer persistent symptoms, including fatigue, fibrotic lung disease, and myalgia; however, the long-term immune response in these patients remains ill-defined. Here, we conducted an observational study examining lymphocyte populations in COVID-19 patients during hospitalization and at up to 6 months of convalescence. We identified a number of lymphocyte alterations that persisted in convalescent patients. Moreover, the compilation of lymphocyte parameters in convalescent COVID-19 patients identified 3 distinct patient subgroups, with 1 subgroup associated with poorer clinical outcome. Our study outlines lymphocyte changes in convalescent COVID-19 patients associated with negative effects on subsequent health.

## Graphical abstract

**Figure:**
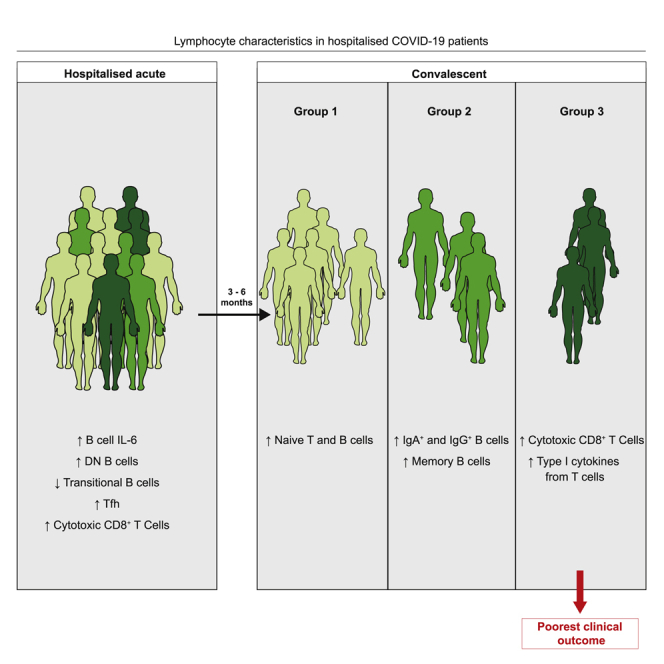

## Teaser

Shuwa et al. examine lymphocyte characteristics in acute and convalescent COVID-19 patients, detailing persistent alterations in lymphocyte phenotype up to 6 months following hospital discharge. In this report, they identify 3 subgroups of convalescent patients based on distinct lymphocyte signatures, with 1 subgroup associated with poorer clinical outcomes.

## Introduction

The coronavirus disease 2019 (COVID-19) pandemic, caused by the emergence of a novel coronavirus strain, has resulted at this time in >106 million infections and 2.3 million deaths worldwide. Infection with severe acute respiratory syndrome-coronavirus-2 (SARS-CoV-2) has a plethora of consequences, ranging from mild influenza-like symptoms to life-threatening and fatal acute respiratory distress syndrome.^1^^,^^2^ In all cases, the pathology is underpinned by not merely the virus itself but also an aberrant inflammatory host immune response. Research efforts detailing immune parameters in patients with acute COVID-19 have significantly improved our understanding of the disease, highlighting profound alterations in the innate and adaptive immune compartments.^3^, ^4^, ^5^, ^6^, ^7^ Lymphopenia and altered lymphocyte function have been reported to correlate with disease severity,^1^^,^^8^ indicating key roles for T and B cells in COVID-19 pathology.

Emerging evidence suggests that COVID-19 patients can develop a spectrum of long-lasting symptoms, including chronic fatigue, myalgia, brain fog, fibrotic lung disease, and pulmonary vascular disease.^9^, ^10^, ^11^ Although the immune response in acute COVID-19 patients has been well characterized, the long-term consequences of SARS-CoV-2 infection remain poorly understood. Since SARS-CoV-2-specific lymphocytes are likely critical for long-term protection against SARS-CoV-2 following disease resolution, it is pivotal to understand their contribution to acute disease, recovery, and long-lasting post-COVID-19 symptoms. Groundbreaking studies are demonstrating that antigen-specific responses to this virus can persist for several months post-infection.^12^, ^13^, ^14^, ^15^ However, given the vast numbers of previously infected individuals across the globe, it is also vital to understand the impact of COVID-19 on the phenotype and functional potential of all lymphocytes, not just those reactive to SARS-CoV2. This will allow for a better understanding of the long-term effects of being hospitalized with COVID-19 on effective immunity. Long-term follow-up of Ebola patients has outlined immune dysfunction persisting for up to 2 years of convalescence.^16^ Following much shorter periods of convalescence, individuals hospitalized with influenza infection have been shown to exhibit continued elevation in CD8^+^ T cell activation and proliferation.^17^ Given the prolonged and profound immune dysregulation seen during acute SARS-CoV2 infection, there is a compelling need to determine whether these alterations translate into longer-term immune alterations and subsequent dysfunction in convalescent individuals.

Here, we examined lymphocytes in COVID-19 patients during hospitalization and in convalescent patients over 6 months following hospital discharge. Specifically, we examined lymphocyte characteristics in peripheral blood mononuclear cells (PBMCs) from blood samples taken from COVID-19 patients within 7 days of hospitalization, at hospital discharge, and at up to 6 months post-hospital discharge. Samples were collected as part of the Coronavirus Immune Response and Clinical Outcomes (CIRCO) study based at 4 hospitals in greater Manchester, UK.^5^ Examination of these samples allowed us to ascertain changes to lymphocytes during acute disease and upon convalescence in COVID-19 patients. We identify key alterations in B cell populations in acute COVID-19 patients with severe disease, which indicate that imbalances within the B cell compartment could contribute to COVID-19 disease severity. Specifically, we demonstrate that in severe COVID-19 patients, there was a loss of transitional B cells and an expansion of double-negative memory B cells. Moreover, B cells exhibited altered functionality with increased production of interleukin-6 (IL-6) during acute disease, which was restored in convalescent patients. Intriguingly, B cell production of IL-10 was higher in convalescent patients with good clinical outcomes compared to patients with poor outcomes. In line with this, we also report changes within the CD4^+^ T cell compartment of acute COVID-19 patients, specifically increases in T follicular helper cells (Tfh) that were recovered in convalescent patients. In contrast, we outline persistent alterations in the functional potential of CD8^+^ T cells, with T cells from convalescent patients exhibiting elevated expression of a cytotoxic program and production of type 1 cytokines. These data describe alterations to lymphocytes, detailing previously undescribed imbalances within the B cell compartment associated with the severity of acute disease and alterations in lymphocyte potential that persist for at least 6 months of convalescence. Furthermore, compiling B and T cell immune parameters from convalescent patients identified 3 patient subgroups, defined by (1) increased cytotoxic T cells and T cell type 1 cytokine production, (2) high proportions of memory, IgA^+^, and IgG^+^ B cells, and (3) the highest expression of trafficking molecules and increased proportions of naive B and T cells. It is noteworthy that the convalescent group defined by the highest proportions of cytotoxic CD8^+^ T cells and type 1 cytokine production was enriched in patients with a poorer outcome at follow-up, defined by abnormal chest X-ray. Our study provides a deeper understanding of lymphocyte responses over the course of COVID-19 and into recovery, highlighting persistent alterations in lymphocyte functionality in convalescent COVID-19 patients up to 6 months following hospital discharge.

## Results and discussion

### Clinical characteristics

Between March 29 and July 15, 2020, we recruited patients during their in-patient stay for COVID-19, who had clinical and lymphocyte data available and who were recruited within 7 days of admission. “Convalescent” patients were recruited between July 14 and October 2020 from outpatient clinical follow-up for COVID-19. Convalescent patients are therefore classified as previously hospitalized COVID-19 patients who are clinically stable and have been discharged from any further inpatient care. Convalescent patients were sampled between 53 and 180 days of convalescence. For convalescent patients sampled twice during their convalescence, they were initially sampled between 67 and 180 days of convalescence and then sampled between 20 and 113 days later (with the latest second sample being taken at day 201 following discharge). For convalescent disease samples, one patient was excluded due to a significant coexisting pathology during inpatient admission for COVID-19; all others were included in the analysis. The median age and overall gender proportions were similar between acute and convalescent groups. There was a larger proportion of severe patients within the convalescent group, reflecting that patients with more severe disease were prioritized for limited face to face appointments in participating trusts. The clinical characteristics of all of the patients recruited to the study are summarized in Tables S1 and S2.

### Altered B cell phenotypes in severe COVID-19 patients are restored upon convalescence

**Figure 1:**
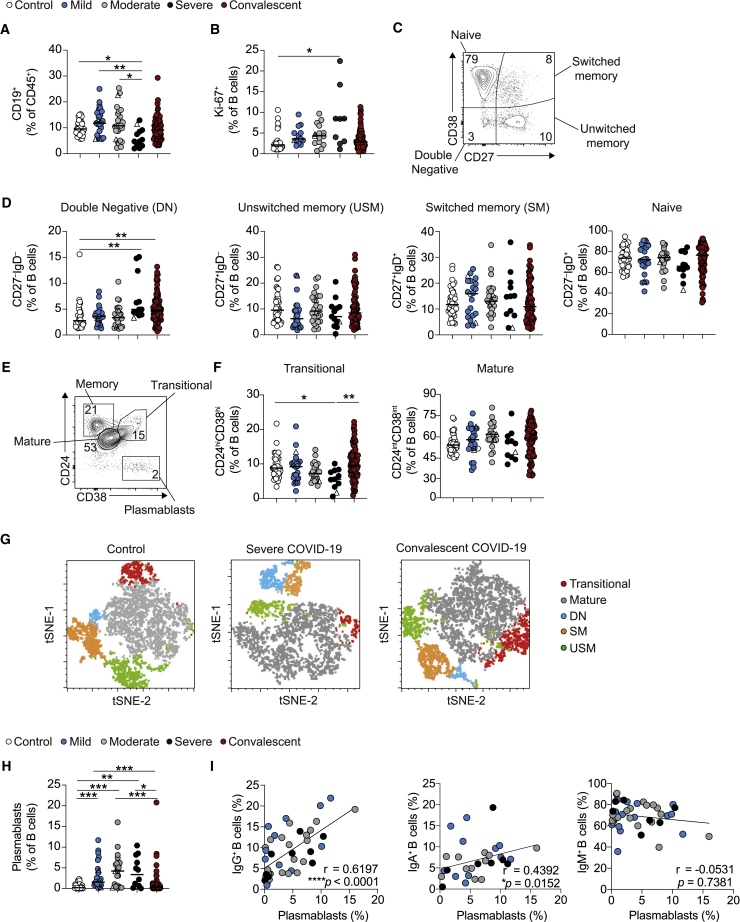
Alterations in B cell subsets during acute COVID-19 are recovered upon convalescence (A) Cumulative data show *ex vivo* frequency of CD19^+^ B cells in healthy individuals (n = 38) and COVID-19 patients with mild (n = 24), moderate (n = 26), and severe (n = 12) disease and at convalescence (n = 83). (B) Cumulative data show Ki-67 expression by B cells in healthy individuals (n = 28) and COVID-19 patients with mild (n = 13), moderate (n = 15), and severe (n = 9) disease and at convalescence (n = 75). (C and D) Representative flow cytometry plots and cumulative data show frequencies of naive (CD27^−^IgD^+^), unswitched memory (CD27^+^IgD^+^), switched memory (CD27^+^IgD^−^), and double-negative (CD27^−^IgD^−^) B cells in healthy individuals (n = 38–40) and COVID-19 with mild (n = 22–24), moderate (n = 25–26), and severe (n = 12–13) disease and at convalescence (n = 78–80). (E and F) Representative flow cytometry plots and cumulative data show *ex vivo* frequency of CD24^hi^CD38^hi^ transitional B cells and CD24^int^CD38^int^ mature B cells in healthy individuals (n = 37) and COVID-19 patients with mild (n = 24), moderate (n = 23), and severe (n = 11) disease and at convalescence (n = 80). (G) tSNE projection of flow cytometry panel visualizing B cell subsets in PBMCs. Representative images for healthy individuals, severe COVID-19 patients, and convalescent patients. Key indicates cell subsets identified on the image. (H) Cumulative data show frequency of CD27^hi^CD38^hi^ plasmablasts in healthy controls (n = 38) and COVID-19 patients with mild (n = 23), moderate (n = 23), and severe (n = 12) disease and at convalescence (n = 81). (I) Graph showing correlation between plasmablasts and IgG^+^ (left), IgA^+^ (center), or IgM^+^ (right) B cell frequencies in acute COVID-19 patients. Graphs show individual patient data, with the bar representing median values. In all graphs, open triangles represent SARS-CoV-2 PCR^−^ patients. ∗p < 0.05, ∗∗p < 0.01, ∗∗∗p < 0.001, 1-way ANOVA with Kruskal-Wallis test with Dunn’s post hoc testing for multiple comparisons or Spearman ranked coefficient correlation test. See also Figures S1 and S2.

In agreement with previously published studies,^5^^,^^6^ we report significantly reduced circulating B cell frequencies in severe COVID-19 patients that were normalized in convalescent patients (Figure 1A). Furthermore, increased Ki-67 expression (indicative of proliferation) in hospitalized patients was not observed in convalescent patients (Figures 1B and S1A). When characterizing B cell subsets based on the expression of CD27 and immunoglobulin D (IgD), we saw an expansion of CD27^−^IgD^−^ double-negative (DN) memory B cells in severe COVID-19 patients that was still present in convalescent patients (Figures 1C and 1D). No differences in unswitched memory (USM), switched memory (SM), or naive B cells were observed between the patient groups and controls. Further classification of B cell subsets into transitional (CD24^hi^CD38^hi^) and mature (CD24^int^CD38^int^) B cells revealed a significant reduction in transitional B cells in patients with severe COVID-19 that was restored in convalescent patients (Figures 1E and 1F). A t-distributed stochastic neighbor embedding (tSNE) representation of the data highlights proportions of subsets in acute and convalescent COVID-19 patients (Figure 1G). Importantly, CD27^hi^CD38^hi^ plasmablasts were the only B cell subset expanded in all COVID-19 patients irrespective of severity, yet proportions were restored in convalescent patients (Figures 1H and S1B). We found that the frequency of plasmablasts positively correlated with the expression of IgG and IgA, but not with IgM expression by B cells in acute COVID-19 patients (Figures 1I, S1C, and S1D), providing further evidence supporting an expansion of class-switched IgA and IgG antibodies in COVID-19 patients.^18^, ^19^, ^20^

Examining B cell phenotypes in individual COVID-19 patients, and tracking them longitudinally from acute hospitalization into convalescence, showed similar alterations in B cell populations. Specifically, we observed a reduction in Ki-67^+^ B cells and plasmablasts at convalescent time points compared to acute disease and a trend toward an increase in transitional B cells (Figure S1E). Interestingly, tracking individual patients from acute disease into convalescence revealed a decrease in DN B cells, despite the global expansion of this subset observed in both severe acute and convalescent patients (Figure S1F). These data highlight alterations in B cell subsets during severe acute COVID-19 that are largely restored upon convalescence.

### Convalescent COVID-19 patients retain phenotypically altered CD8^+^ T cells

**Figure 2:**
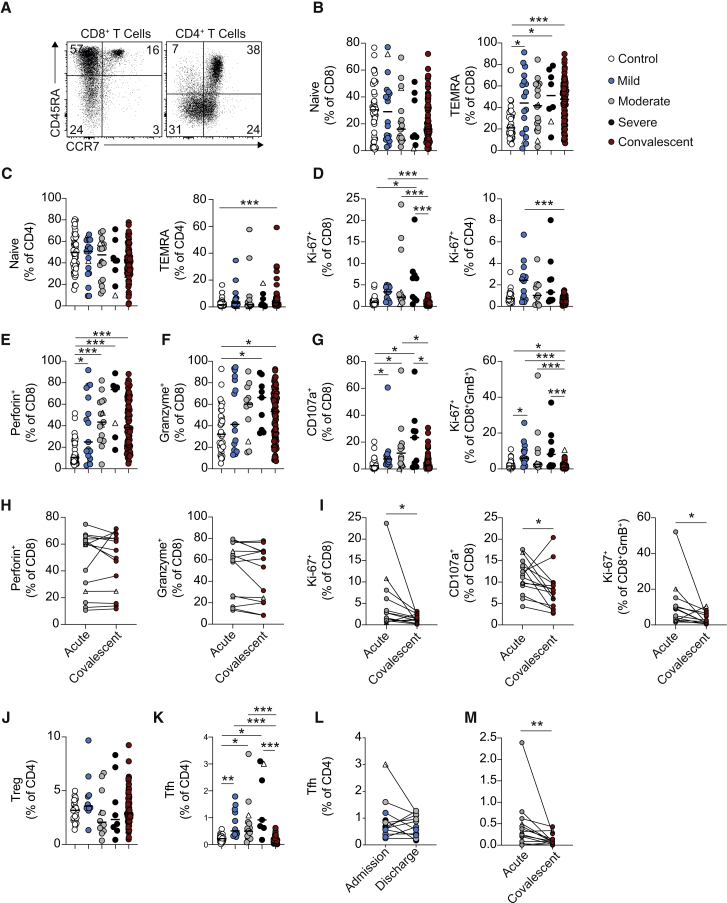
Acute alterations in CD4^+^ T cells and persistent alterations in CD8^+^ T cells during COVID-19 (A) Representative FACS plots showing CD45RA and CCR7 staining on CD4^+^ (gated CD3^+^CD8^−^) and CD8^+^ (gated CD3^+^CD4^−^) T cells. (B and C) Graphs showing frequencies of (B) CD8^+^ and (C) CD4^+^ T cells that have a naive (CD45RA^+^CCR7^+^) and TEMRA (CD45RA^+^CCR7^−^) phenotype in healthy individuals (n = 44) and COVID-19 patients with mild (n = 18–19), moderate (n = 18), and severe (n = 8) disease and at up to 6 months of convalescence (n = 83). (D) Graphs showing frequencies of CD8^+^ and CD4^+^ T cells that stain positive for Ki-67 in healthy individuals (n = 28–30), and COVID-19 patients with mild (n = 14), moderate (n = 11–13), and severe (n = 9) disease and at convalescence (n = 81). (E–G) Graphs showing frequencies of (E) CD8^+^Perforin^+^ cells, (F) CD8^+^GranzymeB^+^ cells, and (G) CD8^+^CD107a^+^ cells and CD8^+^GranzymeB^+^ Ki-67^+^ cells in healthy individuals (n = 29–37), and COVID-19 patients with mild (n = 12–17), moderate (n = 12–15), and severe (n = 7–9) disease and at convalescence (n = 81–83). (H and I) Graphs track frequencies of (H) Perforin^+^ and GranzymeB^+^ and (I) Ki-67^+^, CD107a^+^, and GranzymeB^+^Ki-67^+^CD8^+^ T cells in the same COVID-19 patient at acute (gray circles) and convalescent (maroon circles) time points (n = 14). (J) Graph shows frequencies of Tregs within CD4^+^ T cells of healthy individuals (n = 20) and COVID-19 patients with mild (n = 10), moderate (n = 12), and severe (n = 8) disease and at convalescence (n = 82). (K) Graph shows frequencies of Tfh within CD4^+^ T cells of healthy individuals (n = 34) and COVID-19 patients with mild (n = 12), moderate (n = 15), and severe (n = 7) disease and at convalescence (n = 83). (L) Graph shows frequencies of Tfh in individual acute COVID-19 patients with mild (n = 4), moderate (n = 5), and severe (n = 3) disease at their first and last time points of hospitalization. (M) Graph tracks frequency of Tfh CD4^+^ T cells in the same COVID-19 patient at acute (gray circles) and convalescent (maroon circles) time points (n = 14). Graphs show individual patient data, with the bar representing median values. In all graphs, open triangles represent SARS-CoV-2 PCR^−^ patients. ∗p < 0.05, ∗∗p < 0.01, ∗∗∗p < 0.001, 1-way ANOVA with Kruskal-Wallis test with Dunn’s post hoc testing for multiple comparisons (for B–G and K) or Wilcoxon matched-pairs signed rank test (I and M). See also Figures S1 and S2.

Previous studies have detailed CD8^+^ and CD4^+^ T cell activation in COVID-19 patients.^6^^,^^21^, ^22^, ^23^ Here, we show that convalescent patients exhibited elevated proportions of T effector memory cells positive for CD45RA (TEMRA; CD45RA^+^CCR7^−^) (Figures 2A–2C). Despite this, T cells from convalescent patients did not display elevated expression of the proliferation marker Ki-67 (Figure 2D; see Figures S1G–S1K for representative fluorescence-activated cell sorting (FACS) staining).^6^ CD8^+^ T cells from acute COVID-19 patients exhibit robust expression of a cytotoxic program, with increases in perforin, granzyme, and CD107a expression in unstimulated cells (Figures 2E–2G). Induction of this program was still evident in convalescent patients, whose CD8^+^ T cells exhibited increased expression of both perforin and granzyme (Figures 2E and 2F). However, the proportions of CD107a^+^ and Ki-67^+^GranzymeB^+^CD8^+^ T cells in convalescent patients were reduced compared to those with acute COVID-19, suggesting that cytotoxic CD8^+^ T cells in convalescent patients were no longer actively proliferating or degranulating (Figure 2G). Showing the same pattern, longitudinal tracking of individual patients from their acute time point into convalescence showed unchanged perforin and granzyme B expression in CD8^+^ T cells (Figure 2H), but reduced Ki-67^+^, CD107a^+^, and Ki-67^+^GranzymeB^+^CD8^+^ T cells at convalescent compared to acute time points (Figure 2I). We also noted a persistence of this cytotoxic profile in CD8^+^ T cells in a small subset of convalescent patients examined beyond 6 months after hospital discharge. Obtaining a second convalescent sample from the same patient at a later time point of convalescence demonstrated persistence of this cytotoxic program beyond 6 months (Figure S1L).

In contrast to these phenotypic changes persisting in the CD8^+^ T cell compartment up to, and potentially beyond, 6 months of convalescence, alterations noted in acute disease in CD4^+^ T cells were normalized in convalescent patients. There were no significant changes in regulatory T cells (Tregs) across the disease trajectory (Figure 2J), but during acute disease, there was an expansion in Tfh, defined as CD4^+^CXCR5^+^PD-1^+^ICOS^+^ (Figure 2K). While an increase in Tfh has been reported previously in acute COVID-19 patients,^6^^,^^24^, ^25^, ^26^ we show it was reduced in convalescent patients (Figure 2K). This decrease in Tfh in convalescent patients occurred following hospital discharge (Figure 2L) and could be identified when following the same patient from acute disease into convalescence (Figure 2M). These data demonstrate changes in the functional potential of CD8^+^ T cells up to 6 months following hospital discharge, outlining the continued expression of a cytotoxic program.

### Lymphocytes from acute COVID-19 patients exhibit altered trafficking molecule expression that is restored upon convalescence

Lymphopenia is a well-established hallmark of COVID-19 patients^1^^,^^8^; although the drivers of peripheral blood lymphocyte loss remain unknown, altered trafficking could contribute. Given the importance of appropriate coordination between immune cells during an effective antiviral response and the implications of altered trafficking molecule expression, we examined the expression of chemokine receptors on lymphocytes during acute and convalescent COVID-19. B cells from acute COVID-19 patients displayed a significantly reduced expression of chemokine receptors CXCR3, CXCR5, and the gut homing molecule integrin β7, particularly in patients with more severe disease (Figures S2A–S2D). The expression of CXCR5 and CXCR3 was largely normalized in convalescent patients regardless of acute disease severity (Figure S2E).

Similar to B cells, CXCR5 and CXCR3 expression was substantially reduced in both CD4^+^ and CD8^+^ T cells in acute COVID-19 patients (Figures S2F–S2K), but with β7 exhibiting no alterations in acute disease. This reduction in CXCR3 and CXCR5 occurred irrespective of acute disease severity (Figures S2L and S2M). The loss of CXCR3 and CXCR5 expression was recovered on T cells from convalescent patients (Figures S2F, S2G, S2I, and S2J). In agreement with previous reports examining the expression of CXCR5,^6^ our data outline reduced the expression of multiple trafficking molecules on lymphocytes during acute COVID-19 that are mostly restored upon convalescence. Reduced CXCR3 and CXCR5 expression could reflect reduced homing of lymphocytes into lymph nodes and follicles, which have been reported to contribute to immune dysfunction in other infections, such as advanced HIV.^27^, ^28^, ^29^ Here, we identify changes in chemokine receptor expression during acute disease that are recovered upon convalescence.

### Alterations in lymphocyte cytokine potential in convalescent COVID-19 patients

**Figure 3:**
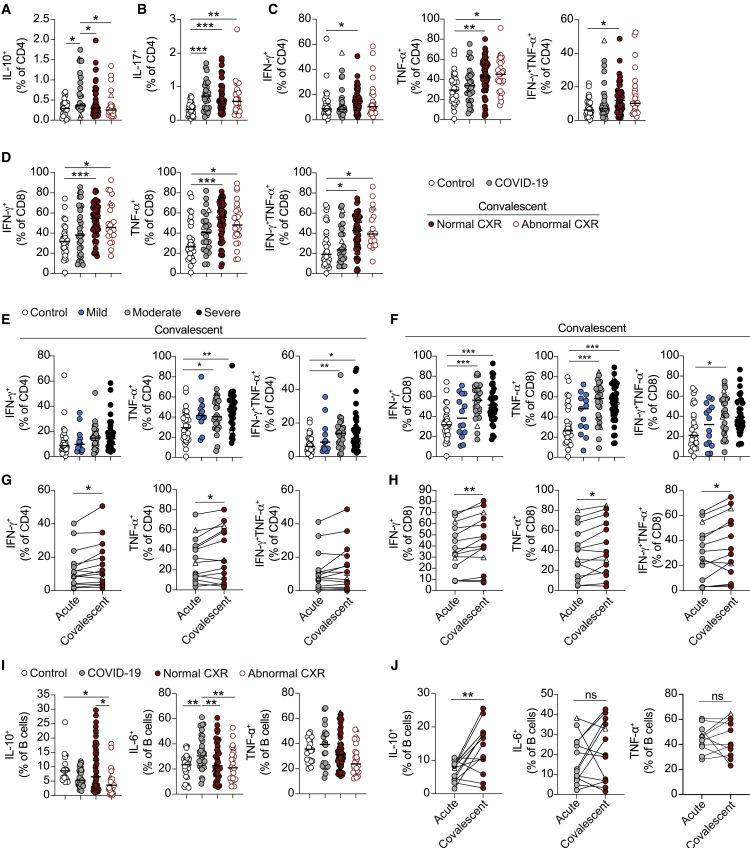
Changes in cytokine production by lymphocytes during acute and convalescent COVID-19 (A–C) Graphs showing frequencies of CD4^+^ T cells that stain positive for (A) IL-10, (B) IL-17, and (C) IFNγ and TNF-α following 3-h stimulation with PMA and ionomycin in healthy individuals (n = 25–30), acute COVID-19 patients (n = 29–33), and convalescent COVID-19 patients with normal (n = 55–57) or abnormal chest X-ray findings (n = 25–26). (D) Graphs showing frequencies of CD8^+^ T cells that stain positive for IFNγ and TNF-α following 3-h stimulation with PMA and ionomycin in healthy individuals (n = 28), acute COVID-19 patients (n = 24–31), and convalescent COVID-19 patients with normal (n = 54–57) or abnormal chest X-ray findings (n = 21–24). (E and F) Graphs show frequencies of (E) CD4^+^ and (F) CD8^+^ T cells that stain positive for IFNγ and TNF-α following 3-h stimulation with PMA and ionomycin in convalescent COVID-19 patients who initially presented with mild (n = 13–14), moderate (n = 25–28), and severe (n = 34–41) disease. (G and H) Graphs track frequencies of (G) CD4^+^ and (H) CD8^+^ T cells that stain positive for IFNγ and TNF-α in the same COVID-19 patient at acute (gray circles) and convalescent (maroon circles) time points (n = 14). (I) Graphs showing frequencies of CD19^+^ B cells positive for IL-10, IL-6, and TNF-α following 48-h stimulation with CpGB in healthy individuals (n = 22–27), acute COVID-19 patients (n = 22–32), and convalescent COVID-19 patients with normal (n = 52–54) or abnormal chest X-ray findings (n = 24–27). (J) Graphs track frequencies of CD19^+^ B cells that stain positive for IL-10, IL-6, and TNF-α in the same COVID-19 patient at acute (gray circles) and convalescent (maroon circles) time points (n = 11–14). Graphs show individual patient data, with the bar representing median values. In all graphs, open triangles represent SARS-CoV-2 PCR^−^ patients. ∗p < 0.05, ∗∗p < 0.01, ∗∗∗p < 0.001, 1-way ANOVA with Kruskal-Wallis test with Dunn’s post hoc testing for multiple comparisons, except for graphs showing CD4^+^TNF-α^+^ and CD8^+^IFNγ^+^ T cells in (E) and (F), where 1-way ANOVA with Holm-Sidak post hoc test was used, or Wilcoxon matched-pairs signed rank test (G, H, and J). See also Figures S3 and S4.

To understand the impact of COVID-19 on the potential of lymphocytes to make distinct cytokines, we stimulated PBMCs with phorbol myristrate acetate (PMA) and ionomycin and examined cytokine production by T cells (see Figure S3A for example staining). Unlike previously published studies probing antigen specificity of T cells in COVID-19 patients,^14^^,^^30^ we more broadly assessed the potential of all T cells in COVID-19 patients to secrete cytokines. This approach allowed us to assess the impact of COVID-19 hospitalization on any subsequent immune response, as opposed to the development of SARS-CoV2-specific memory. IL-10^+^ CD4^+^ T cells were expanded in acute COVID-19 patients, but this was not observed in convalescent patients (Figure 3A). Normalization of IL-10 production from CD4^+^ T cells did not occur during hospitalization, as a decrease in IL-10^+^CD4^+^ T cells was not evident upon discharge (Figure S3B). As previously reported to occur in response to anti-CD3 and anti-CD28 stimulation,^31^ PMA and ionomycin stimulation resulted in increased IL-17^+^CD4^+^ T cells in COVID-19 patients (Figure 3B). Remarkably, enhanced IL-17^+^CD4^+^ T cells persisted into convalescence. We next queried whether the elevated production of IL-17 during convalescence was associated with any specific clinical phenotypes. Increased IL-17^+^CD4^+^ T cells in convalescent patients were seen irrespective of whether patients were stratified by the presentation of normal or abnormal chest X-rays (Figure 3B), reporting versus not reporting increased fatigue, or based upon initial disease severity (Figure S3C).

Despite increased IL-10^+^ and IL-17^+^CD4^+^ T cells, neither CD4^+^ or CD8^+^ T cells from acute COVID-19 patients exhibited altered proportions of cells staining positive for the canonical Th1 cytokines interferon γ (IFNγ) or tumor necrosis factor α (TNF-α) (Figures 3C and 3D). Although a previous study has reported that high proportions of antigen-specific IFNγ T cells are associated with reduced disease severity,^26^ our observation is in line with other studies demonstrating that total peripheral T cell populations exhibit no enhanced production of these cytokines despite ongoing disease^6^^,^^32^ and irrespective of acute disease severity (Figures S3D and S3E). In stark contrast, both CD4^+^ and CD8^+^ T cells from convalescent COVID-19 patients exhibited enhanced production of type 1 cytokines (Figures 3C and 3D). This increase in cytokine production was not evident at hospital discharge (Figures S3F and S3G). Increased type 1 cytokines occurred when stratifying convalescent patients by the presentation of normal or abnormal chest X-rays (Figures 3C and 3D) and those reporting increased fatigue (Figure S3H). Of note, no significant increase in IFNγ^+^CD4^+^ T cells was seen when stratifying patients for fatigue. Increased production of type 1 cytokines in convalescent patients was associated with COVID-19 disease severity, apart from for IFNγ^+^CD4^+^ T cells, as patients who had moderate and severe disease showed significant increases in cytokine-positive cells relative to controls (Figures 3E and 3F). As such, the increases seen in total convalescent patients could be due to increased proportions of patients who exhibited severe disease. However, comparing proportions of cytokine-positive cells in severe patients at acute and convalescent time points still showed an elevation in cytokine-producing T cells, apart from for TNF-α^+^CD8^+^ T cells (Figures S3I and S3J). More important, longitudinal data tracking the same patient across their acute and convalescent disease time points also showed increased production of these type 1 cytokines (except for IFNγ^+^TNF-α^+^CD4^+^ T cells) (Figures 3G and 3H). In a small subset of patients, we also obtained a second convalescent sample after 6 months from hospital discharge. In this small group, we noted unchanged proportions of cytokine-positive T cells, suggesting the persistence of elevated cytokine production beyond 6 months (Figures S3K and S3L). These data fit with previous studies showing IFNγ^+^ and TNF-α^+^ SARS-CoV-2-specific T cells in convalescent patients,^14^^,^^30^^,^^33^ but further outline that altered cytokine potential is a general feature of all T cells during COVID-19 convalescence.

Next, to assess cytokine production by B cells, we stimulated PBMCs from COVID-19 patients with CpGB (a TLR9 agonist) for 48 h and measured cytokine expression by flow cytometry. We observed a significant expansion of IL-6^+^ B cells in acute COVID-19 patients and a trend toward a decrease in IL-10^+^ B cells, suggesting an imbalance in B cells toward a more pro-inflammatory phenotype (Figures 3I and S4A–S4D). The frequency of IL-6^+^ B cells was restored in convalescent patients and was not affected by the presentation of normal or abnormal chest X-rays (Figure 3I) or fatigue (Figure S4D). Interestingly, B cell production of IL-10 was higher in convalescent patients with a good clinical outcome compared to those with a poor outcome. Increased proportions of IL-10^+^ B cells in convalescent patients with normal chest X-rays compared to those with abnormal chest X-rays (Figure 3I) suggests a positive outcome may be associated with the expansion of regulatory B cells. No significant differences in the frequency of TNF-α^+^ B cells were observed in acute and convalescent COVID-19 patients (Figures 3I and S4D). Frequencies of cytokine-positive B cells in both acute and convalescent patients were not affected by disease severity (Figures S4E and S4F). Longitudinal analysis of individual patients from acute disease into convalescence showed no change in either IL-6^+^ or TNF-α^+^ B cells, but it did demonstrate a recovery of IL-10^+^ B cells upon convalescence (Figure 3J). Of note, only 1 of 13 patients whose B cells were followed longitudinally exhibited an abnormal chest X-ray at follow-up. These data demonstrate an expansion of IL-6^+^ B cells during acute disease and reduced proportions of IL-10^+^ B cells in convalescent patients with poor clinical outcomes.

### Identification of COVID-19 convalescent immunotypes based on lymphocyte parameters

**Figure 4:**
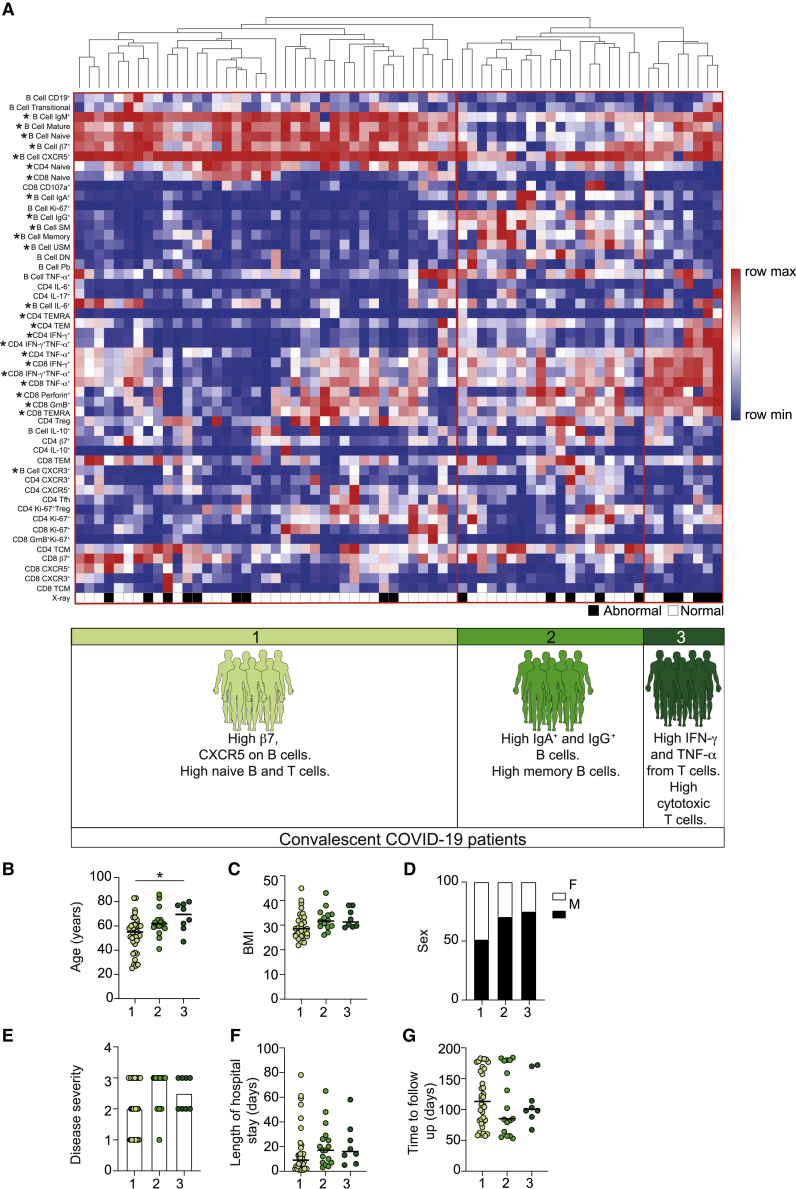
Distinct immune profiles emerge in previously hospitalized convalescent COVID-19 patients (A) Heatmap of indicated immune parameters by row. Each column represents an individual convalescent COVID-19 patient. The patients were clustered using one minus Pearson correlation hierarchical clustering. Significance was determined by 2-way ANOVA, followed by a Tukey’s multiple comparison test. Asterisk next to lymphocyte characteristic indicates a significant difference between patient groups. Dominant immune characteristics of each group are indicated at the bottom of the heatmap. Black and white squares indicate patients displaying a normal (white) or abnormal (black) chest X-ray at follow-up. (B–G) Graphs show patient characteristics and clinical details of convalescent COVID-19 patients in each of the 3 immune groups identified, specifically: (B) age; (C) BMI; (D) sex; (E) severity of acute COVID-19 (with 1 being mild, 2 moderate and 3 severe); (F) length, in days, of hospitalization for acute COVID-19; and (G) time, in days, from hospital discharge to follow-up of convalescent patients. Graphs show individual patient data, with the bar representing median values. ∗p < 0.05, 1-way ANOVA with Kruskal-Wallis test with Dunn’s post hoc testing for multiple comparisons.

Our data establish alterations in the lymphocyte compartment that persist up to 6 months post-hospital discharge in convalescent patients. To further probe lymphocyte changes within convalescent COVID-19 patients, we clustered patients based on T and B cell features. Unsupervised clustering revealed 3 groups of convalescent patients with distinct compositions of lymphocyte signatures (Figure 4A). Group 1 was associated with a high expression of trafficking molecules and increased proportions of naive B and T cells; group 2 was characterized by high proportions of IgA^+^ and IgG^+^ B cells and memory B cells (both switched and unswitched); group 3 displayed increased cytotoxic T cells, CD8^+^ TEMRA, and type 1 cytokines by both CD8^+^ and CD4^+^ T cells. These data suggest the existence of subgroups of convalescent COVID-19 patients based on lymphocyte phenotypes. We next queried whether these groups could identify convalescent patients based on clinical outcome. Higher proportions of patients in group 3 presented with an abnormal chest X-ray and reported breathlessness at their convalescent follow-up (group 1: abnormal chest X-ray, 23.7%, and dyspnea, 43.2%; group 2: abnormal chest X-ray, 26.3%, and dyspnea, 44.4%; group 3: abnormal chest X-ray, 62.5%, and dyspnea, 62.5%). Examining characteristics of the patients within each group (Figures 4B–4G), we noted that group 1 contained most patients who had had mild disease, younger patients, and a greater proportion of female patients, indicating that these parameters could be affecting this convalescent phenotype. In contrast, we noted very little difference in the demographics or acute disease information between patients in groups 2 and 3. For example, both had a similar proportion of patients who had exhibited severe COVID-19 (group 2, 66.6%; group 3, 50%) and similar proportions of males (group 2, 70.5%; group 3, 75%). Despite similar patient characteristics, these 2 patient groups present with distinct lymphocyte profiles and different outcomes, with group 3 exhibiting the poorest clinical outcome (abnormal chest X-ray, group 2, 26.3%; group 3, 62.5%). Our data provide an important foundation for future work supporting the identification of hospitalized COVID-19 patients at risk of developing long COVID symptoms. Our study can now be expanded to explore additional clinical implications of COVID-19, ascertaining whether specific clinical outcomes are associated with distinct convalescent patient subgroups. These consequences can be ascertained as platforms such as the post-hospitalization COVID-19 study (PHOSP-COVID) in the United Kingdom exist to support such future studies. Moreover, any future studies should also explore whether the lymphocyte alterations defined here are specific to COVID-19 convalescence or occur following hospitalization with any respiratory virus.

In summary, here, we report lymphocyte changes across the COVID-19 disease trajectory into convalescence. Our data demonstrate a high degree of activation of a cytotoxic program within CD8^+^ T cells during acute disease, as previously reported.^3^^,^^5^^,^^34^ We extend these data by showing the persistence of this program within circulating CD8^+^ T cells up to at least 6 months of convalescence. Whereas previous studies have shown a persistence of low frequencies of SARS-CoV2-specific CD8^+^ T cells with a cytotoxic profile,^33^ importantly, here, we show the elevation of cytotoxic markers within total circulating CD8^+^ T cells. Although the specificity of T cells in our study remains to be determined, increases in CD8^+^ T cells with a cytotoxic potential would result in an altered T cell landscape that could affect tissue integrity, depending on their trafficking capabilities and cytokine responsiveness. Moreover, our data raise questions about the impact that this increase in cytotoxic cells would have on subsequent infection, be that bacterial, viral, or fungal. The importance of this question is further underscored by the elevated potential of total CD8^+^ and CD4^+^ T cells to produce IFNγ and TNF-α in convalescent patients, outlining a persistent alteration in cytokine potential that could be either beneficial or detrimental to subsequent immune responses.

In contrast, altered B cell subsets in acute disease are recovered upon convalescence. Several recent studies have reported the detection of virus-specific antibodies for several months post-recovery from SARS-CoV-2 infection.^35^, ^36^, ^37^ Here, we show that circulating plasmablast frequencies correlate positively with IgA/IgG and negatively with IgM, further supporting an expansion of class-switched antibodies in COVID-19 patients. In addition to secreting antibodies, B cells produce cytokines and are classified into effector (Beff; IL-6^+^ and TNF-α^+^) and regulatory B cell (Breg; IL-10^+^) subsets based on the cytokines that they produce.^38^ Significant decreases in transitional B cells, the precursors of human Bregs,^39^ along with IL-10^+^ B cells, suggest a loss of immunosuppressive Bregs and an expansion of Beff cells in severe COVID-19 patients, as also observed in chronic inflammatory disorders such as systemic lupus erythematosus (SLE) and rheumatoid arthritis (RA).^39^, ^40^, ^41^ Interestingly, the resolution of lung pathology in COVID-19 patients was found to be associated with higher proportions of IL-10^+^ B cells, suggesting that these cells could be important in suppressing excess inflammation, and are associated with positive long-term outcomes.

In summary, we report phenotypic and functional alterations to B and T cells across the trajectory of SARS-CoV-2 responses from acute disease requiring hospitalization into convalescence, identifying immune alterations that persist in convalescent COVID-19 patients for up to 6 months. Our study therefore identifies lymphocyte changes in convalescent COVID-19 patients, which could have longer-term effects on subsequent anti-pathogen or auto-inflammatory responses.

### Limitations of study

Our study has limitations, including the reduced number of samples from patients at acute disease (n = 58) relative to those during convalescence (n = 83); in fact, for some parameters, only 30 acute samples were examined. Furthermore, disease severity in the acute group did not match that in the convalescent group; the latter contained more severe patients (acute = 22.4%; convalescence = 49.4%). In addition, only 14 patients were followed longitudinally from acute disease into convalescence. Our study would have been significantly enhanced if we could have followed more individual patients. Our study would have been further enhanced were it possible to examine convalescent patients at later time points—for example, 9 months and 1 year.

## Consortia

The members of CIRCO are Rohan Ahmed, Miriam Avery, Katharine Birchall, Evelyn Charsley, Alistair Chenery, Christine Chew, Richard Clark, Emma Connolly, Karen Connolly, Simon Dawson, Laura Durrans, Hannah Durrington, Jasmine Egan, Kara Filbey, Claire Fox, Helen Francis, Miriam Franklin, Susannah Glasgow, Nicola Godfrey, Kathryn J. Gray, Seamus Grundy, Jacinta Guerin, Pamela Hackney, Chantelle Hayes, Emma Hardy, Jade Harris, Anu John, Bethany Jolly, Verena Kästele, Gina Kerry, Sylvia Lui, Lijing Lin, Alex G. Mathioudakis, Joanne Mitchell, Clare Moizer, Katrina Moore, Stuart Moss, Syed Murtuza Baker, Rob Oliver, Grace Padden, Christina Parkinson, Michael Phuycharoen, Ananya Saha, Barbora Salcman, Nicholas A. Scott, Seema Sharma, Jane Shaw, Joanne Shaw, Elizabeth Shepley, Lara Smith, Simon Stephan, Ruth Stephens, Gael Tavernier, Rhys Tudge, Louis Wareing, Roanna Warren, Thomas Williams, Lisa Willmore, and Mehwish Younas.

## STAR★Methods

### Key resources table

**Table:** 

REAGENT or RESOURCE	SOURCE	IDENTIFIER
**Antibodies**		

Human TruStain FcX (Fc Receptor Blocking solution) (5ul/stain)	Biolegend	Cat# 422302; RRID: AB_2818986
PE-Cyanine7 anti-human CD19 (clone: HIB19, 1 in 50)	Biolegend	Cat# 302216; RRID: AB_314246
PE-Dazzle 594 anti-human CD27 (clone: M-T271, 1 in 50)	Biolegend	Cat# 356422; RRID: AB_2564101
PerCP-Cyanine 5.5 anti-human CD38 (clone: HIT2, 1 in 50)	Biolegend	Cat# 303522; RRID: AB_893314
Brilliant Violet 605 anti-human IgD (clone: IA6-2, 1 in 50)	Biolegend	Cat# 348232; RRID: AB_2563337
Brilliant Violet 421 anti-human IgG (clone: M1310G05, 1 in 50)	Biolegend	Cat# 410704; RRID: AB_2565626
Brilliant Violet 510 anti-human IgM (clone: MHM-88, 1 in 50)	Biolegend	Cat# 314522; RRID: AB_2562916
APC anti-human IgA (clone: REA1014, 1 in 100)	Miltenyi Biotech	Cat# 130-116-879; RRID: AB_2727739
Brilliant Violet 785 anti-human CD11c (clone: 3.9, 1 in 50)	Biolegend	Cat# 301644; RRID: AB_2565779
PE anti-human CD86 (clone: BU63, 1 in 50)	Biolegend	Cat# 374206; RRID: AB_2721633
PE anti-human IL-6 (clone: MQ2-13A5, 1 in 100)	Biolegend	Cat# 501107; RRID: AB_315155
APC anti-human IL-10 (clone: JES3-19F1, 1 in 25)	Biolegend	Cat# 506807; RRID: AB_315457
Brilliant Ultra Violet 395 anti-human TNFa (clone: Mab11, 1 in 50)	BD Biosciences	Cat# 563996; RRID: AB_2738533
Alexa Fluor 700 anti-human IL-17 (clone: BL168, 1 in 50)	Biolegend	Cat# 512318; RRID: AB_2124868
PE-Cyanine7 anti-human IFNg (clone: 4S.B3, 1 in 50)	Biolegend	Cat# 502528; RRID: AB_2123323
Alexa Fluor 700 anti-human Ki-67 (clone: Ki-67, 1 in 50)	Biolegend	Cat# 350530; RRID: AB_2564040
Brilliant Violet 510 anti-human CXCR3 (clone: G025H7, 1 in 50)	Biolegend	Cat# 353726; RRID: AB_2563642
APC anti-human CXCR5 (clone: J252D4, 1 in 50)	Biolegend	Cat# 356907; RRID: AB_2561816
Brilliant Violet 605 anti-human b7 (clone: FIB504, 1 in 50)	BD Biosciences	Cat# 564284; RRID: AB_2738729
Brilliant Violet 650 anti-human b7 (clone: FIB504, 1 in 50)	BD Biosciences	Cat# 564285; RRID: AB_2738730
Alexa Fluor 488 anti-human Blimp1 (clone: 646702, 1 in 25)	R&D Systems	Cat# IC36081G; RRID: AB_11129439
APC-eFluor 780 anti-human CD24 (clone: eBioSN3, 1 in 50)	eBioscience	Cat# 47-0247-42; RRID: AB_10735091
FITC anti-human CD56 (clone: 5.1H11, 1 in 50)	Biolegend	Cat# 362546; RRID: AB_2565964
Brilliant Violet 650 anti-human CD3 (clone: OKT3, 1 in 50)	Biolegend	Cat# 317324; RRID: AB_2563352
Brilliant Violet 605 anti-human CD3 (clone: OKT3, 1 in 50)	Biolegend	Cat# 317322; RRID: AB_2561911
Brilliant Ultra Violet 395 anti-human CD3 (clone: SK7, 1 in 50)	BD Biosciences	Cat# 564001; RRID: AB_2744382
PerCP-Cyanine 5.5 anti-human CD4 (clone: SK3, 1 in 50)	Biolegend	Cat# 344608; RRID: AB_1953235
Alexa Fluor 700 anti-human CD4 (clone: SK3, 1 in 50)	Biolegend	Cat# 344622; RRID: AB_2563150
Brilliant Violet 510 anti-human CD8 (clone: SK1, 1 in 50)	Biolegend	Cat# 344732; RRID: AB_2564624
Brilliant Violet 785 anti-human CD8 (clone: SK1, 1 in 50)	Biolegend	Cat# 344740; RRID: AB_2566202
PE-Dazzle 594 anti-human TCRgd (clone: B1, 1 in 50)	Biolegend	Cat# 331226; RRID: AB_2565534
Alexa Fluor 488 anti-human ICOS (clone: C398.4A, 1 in 50)	Biolegend	Cat# 313514; RRID: AB_2122584
PE anti-human PD-1 (clone: EH12.2H7, 1 in 50)	Biolegend	Cat# 329906; RRID: AB_940483
APC-Cyanine7 anti-human CCR7 (clone: G043H7, 1 in 50)	Biolegend	Cat# 353212; RRID: AB_10916390
Brilliant Violet 605 anti-human CD45RA (clone: HI100, 1 in 50)	Biolegend	Cat# 304134; RRID: AB_2563814
Brilliant Violet 711 anti-human CD25 (clone: BC96, 1 in 50)	Biolegend	Cat# 302636; RRID: AB_2562910
Brilliant Violet 785 anti-human CD127 (clone: A019D5, 1 in 50)	Biolegend	Cat# 351330; RRID: AB_2563605
PE-Dazzle 594 anti-human CD107a (clone: H4A3, 1 in 100)	Biolegend	Cat# 328646; RRID: AB_2566115
Brilliant Violet 421 anti-human GranzymeB (clone: QA18A28, 1 in 50)	Biolegend	Cat# 396414; RRID: AB_2810603
PE-Cyanine7 anti-human Perforin (clone: dG9, 1 in 50)	Biolegend	Cat# 308126; RRID: AB_2572049
Alexa Fluor 488 anti-human Foxp3 (clone: 236A/E7, 1 in 50)	eBioscience	Cat# 53-4777-42; RRID: AB_10804652

**Biological samples**		

Blood samples (hospitalised patients at acute time point)	UK, This paper	Table S1
Blood samples (hospitalised patients at 3-8 months convalescence)	UK, This paper	Table S2

**Chemicals, peptides, and recombinant proteins**		

Foxp3/transcription Factor staining buffer Set	ThermoFisher	00-5523-00
Brefeldin A	Biolegend	420601
Stimulation cocktail	ThermoFisher	00-4970-03
Zombie UV Fixable Viability kit	Biolegend	423108
Zombie NIR Fixable Viability kit	Biolegend	423106
UltraComp eBeads	ThermoFisher	01-2222-42
PBS	GIBCO	10010023
RPMI 1640	GIBCO	31870025
L-glutamine	GIBCO	A2916801
HEPES	GIBCO	15630056
MEM Non-essential Amino Acids	GIBCO	11140050
Penicillin-Streptomycin	GIBCO	15070063
Beta-mercaptoethanol	GIBCO	31350-010
FBS	ThermoFisher	10500064
CpG (ODN 2006)	Cambridge Bioscience	HC4039-200NMOL
Ficoll Plaque Plus	GE Healthcare	GE17-1440-03

**Software and algorithms**		

FlowJo	Treestar	https://www.flowjo.com/
GraphPad Prism	GraphPad Software	https://www.graphpad.com/

**Other**		

SepMATE tubes - 50	StemCell Technologies	85460

### Resource availability

#### Lead contact

Further information and requests for resources and information should be directed to the Lead Contact, Joanne E. Konkel (Joanne.konkel@manchester.ac.uk).

#### Materials availability

This study did not generate new unique reagents.

#### Data and code availability

All relevant data outputs are within the paper and supplemental information.

### Experimental models and subject details

#### Study design and participants

Two cohorts of patients were recruited from Manchester University Foundation Trust (MFT), Salford Royal NHS Foundation Trust (SRFT) and Pennine Acute NHS Trust (PAT) under the framework of the Manchester Allergy, Respiratory and Thoracic Surgery (ManARTS) Biobank (study no M2020-88) for MFT or the Northern Care Alliance Research Collection (NCARC) tissue biobank (study no NCA-009) for SRFT and PAT. Ethical approval obtained from the National Research Ethics Service (REC reference 15/NW/0409 for ManARTS and 18/WA/0368 for NCARC). Informed consent was obtained from each patient, clinical information was extracted from written/electronic medical records including demographic data, presenting symptoms, comorbidities, radiographic findings, vital signs, and laboratory data. Patients were included if they tested positive for SARS-CoV-2 by reverse-transcriptase–polymerase-chain-reaction (RT-PCR) on nasopharyngeal/oropharyngeal swabs or sputum during their in-patient admission for COVID-19. Patients with negative nasopharyngeal RTPCR results were also included if there was a high clinical suspicion of COVID-19, the radiological findings supported the diagnosis, and there was no other explanation for symptoms. Patients were excluded if an alternative diagnosis was reached, where indeterminate imaging findings were combined with negative SARS-CoV-2 nasopharyngeal (NP) test, or there was another confounding acute illness not directly related to COVID-19. The severity of disease was scored each day, based on criteria for escalation of care (Table S3). Where severity of disease changed during admission, the highest disease severity score was selected for classification. Peripheral blood samples were collected within 7 days of hospital admission, at discharge and then at 3-9 months post hospital discharge when patients returned to out-patient clinics.

Demographics and clinical information for acute and convalescent patients can be found in Tables S1 and S2.

#### Healthy controls

Recruiting healthy individuals from the community for blood sampling during the SARS-CoV-2 outbreak was not possible, and therefore we sampled frontline workers from NHS Trusts and University of Manchester staff with an age range that was similar to our COVID-19 patients (age range 35-71; median age = 50.9; 52% males). All healthy controls tested negative for anti-Spike1 receptor binding domain antibodies.

### Method details

#### PBMC isolation

Fresh blood samples from COVID-19 patients and healthy individuals were collected in EDTA tubes. Blood was diluted 1:1 with PBS and layered gently on Ficoll-Paque in SepMATE tubes (StemCell Technologies) followed by density gradient centrifugation. Cells were thoroughly washed and were either freshly stained for flow cytometry or are stored in 10% dimethyl sulfoxide (DMSO) in fetal bovine serum (FBS) at −150°C.

#### Cell culture

Frozen PBMC were thawed, washed and resuspended in RPMI containing 10% FBS, L-Glutamine, non-essential amino acids, HEPES, and penicillin plus streptomycin. 2.5 × 10^5^ cells were stimulated with; (i) 2μL/ml of stimulation cocktail (eBioScience) in the presence of 10 μg/ml Brefeldin A for three hours (T cells), (ii) 1 μM CpG for 48 hours followed by 2μL/ml of stimulation cocktail (eBioScience) in the presence of 10 μg/ml Brefeldin A in the last four hours (B cells). Following stimulation cells were washed and stained for flow cytometric analysis.

#### Flow cytometry

PBMCs (fresh/thawed/stimulated) were stained with fluorophore conjugated antibodies (see Key resources table) and viability dyes. Samples were acquired on an LSRFortessa cell analyzer (Becon Dickinson) and analyzed using FlowJo (TreeStar).

### Quantification and statistical analysis

#### Clustering of T and B cell phenotypes

T cell and B cell data were scaled using unit variance scaling, clustered and graphed using correlation distance and average linkage on the heatmap tool on ClustVis.^42^

#### Statistics

Results are presented as individual data points with medians. Normality tests were performed on all datasets. Groups were compared using an unpaired Mann-Whitney test for healthy individuals versus COVID-19 patients, one-way ANOVA with Holm-Sidak post hoc testing (normal distribution) or Kruskal-Wallis test with Dunn’s post hoc testing (failing normality testing) for multiple comparisons, or Spearman’s rank correlation coefficient test for correlation of separate parameters within the COVID-19 patient group, using Prism 8 software (GraphPad). In all cases, a p value of ≤ 0.05 was considered significant. Where no statistical difference is shown there was no significant difference. Details of statistical tests and definitions of n can be found in each figure legend.

## Supplemental information

Document S1. Tables S1–S3 and Figures S1–S4

Document S2. Article plus supplemental information
