# Supplementary material for: Alterations in T and B cell function persist in convalescent COVID-19 patients
Source: Med. 2021 Jun 11;2(6):720–735.e4. doi: 10.1016/j.medj.2021.03.013 (PMC8011689; doi:10.1016/j.medj.2021.03.013)
Supplement: Document S1. Tables S1–S3 and Figures S1–S4 [file mmc1.pdf]

Med, Volume *2*

Supplemental information

Alterations in T and B cell function persist in convalescent COVID-19 patients

Halima A. Shuwa, Tovah N. Shaw, Sean B. Knight, Kelly Wemyss, Flora A. McClure, Laurence Pearmain, Ian Prise, Christopher Jagger, David J. Morgan, Saba Khan, Oliver Brand, Elizabeth R. Mann, Andrew Ustianowski, Nawar Diar Bakerly, Paul Dark, Christopher E. Brightling, Seema Brij, CIRCO, Timothy Felton, Angela Simpson, John R. Grainger, Tracy Hussell, Joanne E. Konkel, and Madhvi Menon

Supplemental Information

Alterations in T and B cell function persist in convalescent COVID-19 patients

Halima A. Shuwa, Tovah N. Shaw, Sean B. Knight, Kelly Wemyss, Flora A. McClure, Laurence Pearmain, Ian Prise, Christopher Jagger, David. J. Morgan, Saba Khan, Oliver Brand, Elizabeth R. Mann, Andrew Ustianowski, Nawar Diar Bakerly, Paul Dark, Christopher E. Brightling, Seema Brij, CIRCO, Timothy Felton, Angela Simpson, John R. Grainger, Tracy Hussell, Joanne E. Konkel, Madhvi Menon.

Supplemental Table 1. Clinical characteristics of acute COVID-19 patients, Related to Figures 1-4**.** Data are median (IQR)^{m} , where^{m} is the number of missing data points, n (%) or n/N (%), where N is the total number with available data. PE, pulmonary embolism, AKI, Acute kidney injury. ^{a} Admission observations.

Acute Patients

	**Overall (58)**	**Mild (22)**	**Moderate (23)**	**Severe (13)**
Age	58.5 (49.5-71)	58 (45-71)	58 (50-66.5)	67 (55-74)
**Gender**				
Male	36/58 (62.1%)	13/22 (59.1%)	13/23 (56.5%)	10/13 (77%)
Female	22/58 (37.9%)	9/22 (40.9%)	10/23 (43.5%)	3/13 (23%)
BMI	28.5 (25.2-30.6)^{14}	27.8 (23.2-33.1)^{8}	29 (26.8-30.3)^{3}	27.6 (25.4-30.2)^{3}
Day of admission recruited	2 (2-3)	3 (2-4.75)	2 (2-3)	2 (2-3)
**Medical History**				
Diabetes	13/58 (22.41%)	6/22 (27.27%)	2/23 (8.7%)	5/13 (38.46%)
Ischaemic Heart Disease	8/58 (13.79%)	3/22 (13.64%)	2/23 (8.7%)	3/13 (23.08%)
Hypertension	20/58 (34.48%)	6/22 (27.27%)	9/23 (39.13%)	5/13 (38.46%)
COPD	11/58 (18.97%)	5/22 (22.73%)	4/23 (17.39%)	2/13 (15.38%)
Asthma	9/58 (15.52%)	4/22 (18.18%)	4/23 (17.39%)	1/13 (7.69%)
Malignancy	5/58 (8.6%)	0/22 (0%)	2/23 (8.7%)	3/13 (23%)
**Differential counts on admission (x 10^{9}/L)**				
Total white cell count	7.1 (6-10)^{9}	6.85 (5.55-8.42)^{4}	7.3 (6.3-10)^{2}	7.25 (6.55-10.02)^{3}
Lymphocytes	1 (0.82-1.5)^{9}	1.2 (0.92-1.77)^{4}	0.9 (0.8-1.5)^{2}	0.9 (0.82-0.98)^{3}
Neutrophils	5.3 (4.4-8)^{9}	5.15 (3.8-7.05)^{4}	5.5 (4.7-7.7)^{2}	6.25 (4.82-8.62)^{3}
Monocytes	0.4 (0.2-0.6)^{9}	0.25 (0.2-0.73)^{4}	0.5 (0.4-0.7)^{2}	0.35 (0.3-0.57)^{3}
**Other inpatient investigations**				
Positive SARS CoV2 PCR	49/58 (84%)	18/22 (81%)	20/23 (87%)	11/13 (85%)
Highest CRP (mg/L)	144 (83.9-226)	99 (35-176)	131 (86.5-195.6)	256 (198-283)
**Chest Imaging**				
Bilateral opacification	49/56 (87.5%)	14/20 (70%)	22/23 (95.7%)	13/13 (100%)
Unilateral opacification	3/56 (5.4%)	2/20 (10%)	1/23 (4.3%)	0/13 (0%)
Clear	4/56 (7.1%)	4/20 (20%)	0/23 (0%)	0/13 (0%)
**Outcome**				
Length of stay (days)	6 (4-11.3)^{2}	5.5 (3.3-9.2)	6 (4-10)^{2}	10 (6-13)
Mortality	9/58 (15.5%)	1/22 (4.5%)	0/23 (0%)	8/13 (61.5%)

Supplemental Table 2. Clinical characteristics of convalescent COVID-19 patients Related to Figures 1-4. Data are median (IQR)^{m} , where^{m} is the number of missing data points, n (%) or n/N (%), where N is the total number with available data. PE, pulmonary embolism, AKI, Acute kidney injury. ^{a} Admission observations.

Convalescent Patients

	**Overall (83)**	**Mild (15)**	**Moderate (27)**	**Severe (41)**
Age (years)	60 (51 - 66.5)	51 (45 - 59.54)	60 (51.5 - 71)	60 (55.63 - 66)
**Gender**				
Male	51/83 (61.4%)	10/15 (66.7%)	14/27 (51.9%)	27/41 (65.9%)
Female	32/83 (38.6%)	5/15 (33.3%)	13/27 (48.1%)	14/41 (34.1%)
BMI	31 (28.7 - 34.7)^{31}	34.8 (31.9 - 38.4)^{5}	30.9 (28.8 - 34.6)^{13}	29.6 (28.5 - 33.1)^{13}
**Medical History**				
Diabetes	21/83 (25.3%)	4/15 (26.67%)	9/27 (33.33%)	8/41 (19.51%)
Ischaemic heart disease	17/83 (20.48%)	2/15 (13.33%)	12/27 (44.44%)	3/41 (7.32%)
Hypertension	32/83 (38.55%)	3/15 (20%)	13/27 (48.15%)	16/41 (39.02%)
COPD	14/83 (16.87%)	3/15 (20%)	9/27 (33.33%)	2/41 (4.88%)
Asthma	30/83 (36.14%)	7/15 (46.67%)	9/27 (33.33%)	14/41 (34.15%)
Malignancy	5/83 (6.02%)	0/15 (0%)	3/27 (11.1%)	2/41 (4.9%)
**ACUTE ADMISSION**				
**Differential blood counts on acute admission ($x10^{9}/L$)**				
Total white cell count	7.3 (5.77 - 10.1)^{11}	6.1 (4.8 - 7.5)^{2}	6.65 (5.63 - 9.55)^{7}	8.7 (6.8 - 11.35)^{2}
Lymphocytes	0.89 (0.7 - 1.21)^{11}	1.1 (0.86 - 1.63)^{2}	0.98 (0.73 - 1.42)^{7}	0.8 (0.62 - 0.95)^{2}
Neutrophils	5.99 (4.13 - 8.75)^{11}	4.04 (3.7 - 5.7)^{2}	4.81 (4.04 - 7.68)^{7}	7.03 (5.34 - 10.2)^{2}
Monocytes	0.4 (0.27 - 0.56)^{11}	0.27 (0.23 - 0.55)^{2}	0.48 (0.3 - 0.6)^{7}	0.4 (0.3 - 0.52)^{2}
**Other inpatient investigations**				
Positive SARS CoV2 test	78/83 (94%)	15/15 (100%)	25/27 (92.6%)	38/41 (92.7%)
Highest CRP (mg/L)	135 (66 - 245.5)^{3}	50 (26.5 - 88.5)	113 (70 - 151.5)^{1}	256 (131 - 330)^{2}
**Chest imaging**				
Bilateral opacification	59/64 (92.1%)	7/9 (77.8%)	21/23 (91.3%)	31/32 (96.9%)
Unilateral opacification	4/64 (6.3%)	1/9 (11.1%)	2/23 (8.7%)	1/32 (3.1%)
Clear	1/64 (1.6%)	1/9 (11.1%)	0/23 (0%)	0/32 (0%)
**Outcome**				
Length of stay (days)	11 (5.5 – 21.5)	2 (1 - 5.5)	7 (4.5 - 13)	20 (13 - 39)
**CONVALESCENCE**				
Admission to follow up (days)	158 (116.5 - 184.5)	174 (140.5 - 185)	140 (105 - 171.5)	155 (126 - 192)
Discharge to follow up (days)	131 (90 - 178)	169 (138.5 - 181.5)	125 (95.5 - 165)	117 (87 - 180)
**Symptoms and investigations at follow up**				
Dyspnoea	38/82 (46.34%)	6/15 (40%)	16/27 (59.26%)	16/40 (40%)
Resolved CXR	55/83 (66.3%)	12/15 (80%)	19/27 (70.4%)	24/41 (58.5%)
Persistent CXR features	28/83 (33.7%)	3/15 (20%)	8/27 (29.6%)	17/41 (41.5%)

Supplemental Table 3. Patient categorisation Information Related to Figures 1-4**.** Criteria for patient stratification. NIV, non-invasive ventilation; CPAP, continuous positive airway pressure; ICU, intensive care.

**Severity Score**	**Criteria**
**Mild**	- <3l or 28% supplemental oxygen required to maintain oxygen saturations.<br>- Managed in a ward based environment.
**Moderate**	- Breathless<br>- <10l or <60% supplemental oxygen required to maintain oxygen saturations.<br>- Managed in a ward based environment.<br>- Chronic NIV or CPAP (home use) or acute NIV for COPD.
**Severe**	Any of:<br>- >10l or 60% supplemental oxygen required to maintain oxygen saturations.<br>- Use of acute NIV (not for COPD)<br>- Managed in ICU/invasive ventilation.

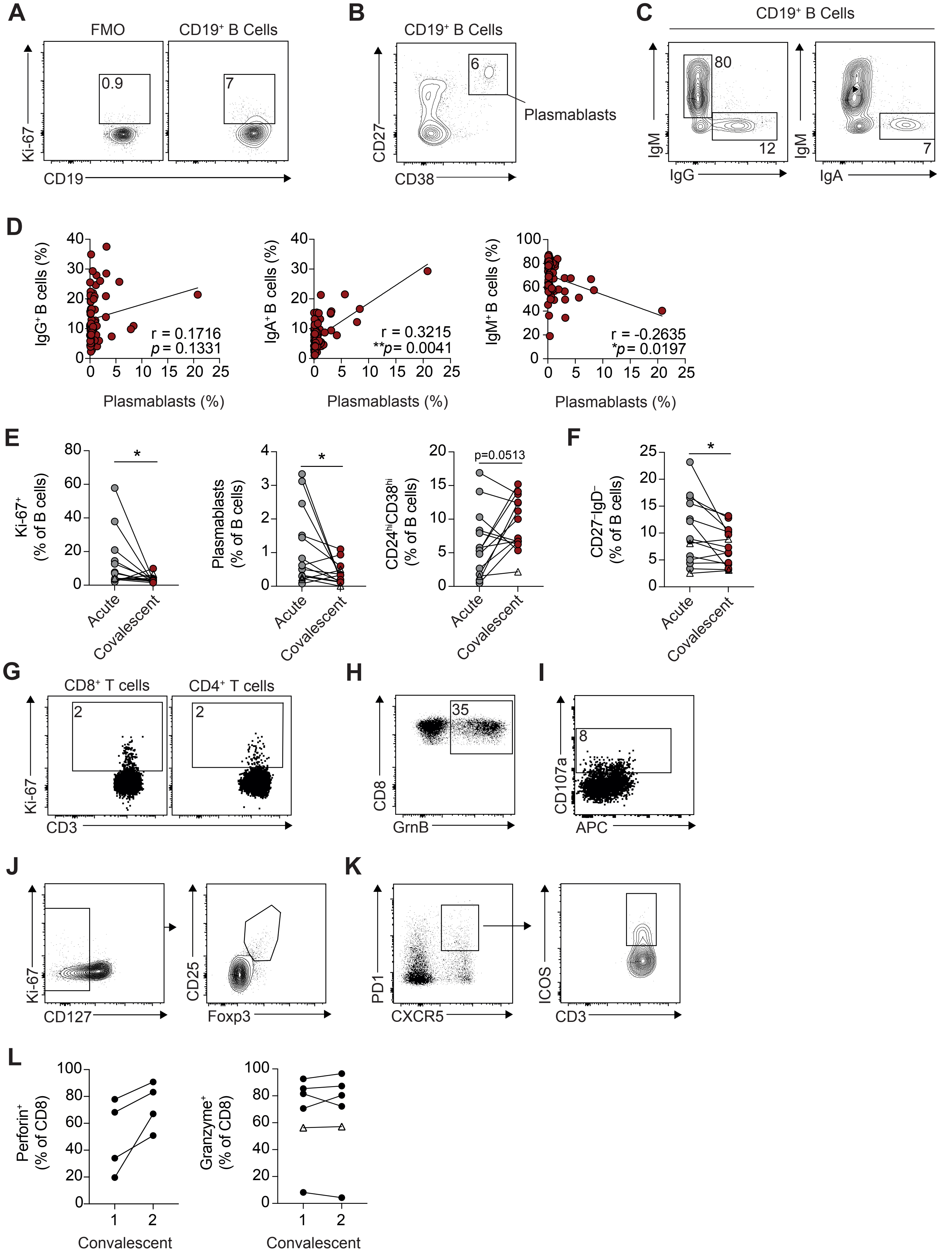

Supplementary Figure 1

Supplemental Figure 1. T and B cell subsets in hospitalized COVID-19 patients, Related to Figures 1 and 2. **(A)** Representative FACS plots showing Ki-67 staining on $CD19^+$ B cells. **(B)** Representative FACS plots showing the gating strategy for $CD27^{hi}CD38^{hi}$ plasmablasts. **(C)** Representative FACS plots gated on $CD19^+$ B cells staining positive for IgG, IgM or IgA.**(D)** Graphs showing correlation between plasmablasts and IgG^+ (left), IgA^+ (middle) or IgM^+ (right) B cell frequencies in convalescent COVID-19 patients (n=78) . **(E,F)** Graphs track frequencies of **(E)** $Ki67^+$ B cells, plasmablasts, transitional ($CD24^{hi}CD38^{hi}$) B cells, and **(F)** $CD27^-IgD^-$ B cells in the same COVID-19 patient at acute (grey circles) and convalescent (maroon circles) time-points (n=14). (**G**) Representative FACS plots showing Ki-67 staining on $CD4^+$ and $CD8^+$ T cells. **(H,I)** Representative FACS plots showing $CD8^+$ T cells staining positive for **(H)** GranzymeB and **(I)** CD107a. **(J)** Representative FACS plots gated on $CD3^+CD4^+CD127^{lo/neg}$ T cells staining positive for CD25 and foxp3. **(K)** Representative FACS plots showing gating to identify Tfh cells ($CD3^+CD4^+$PD- 1^+CXCR5^+ $ICOS^+$). **(L)** Graphs track frequencies of $CD8^+$ T cells which are $perforin^+$ and $granzymeB^+$ in the same COVID-19 patient at convalescent time-points pre (1) and post (2) 6 months since hospital discharge (n=4-6). In all graphs, triangles represent SARS-CoV-2 PCR-negative patients.

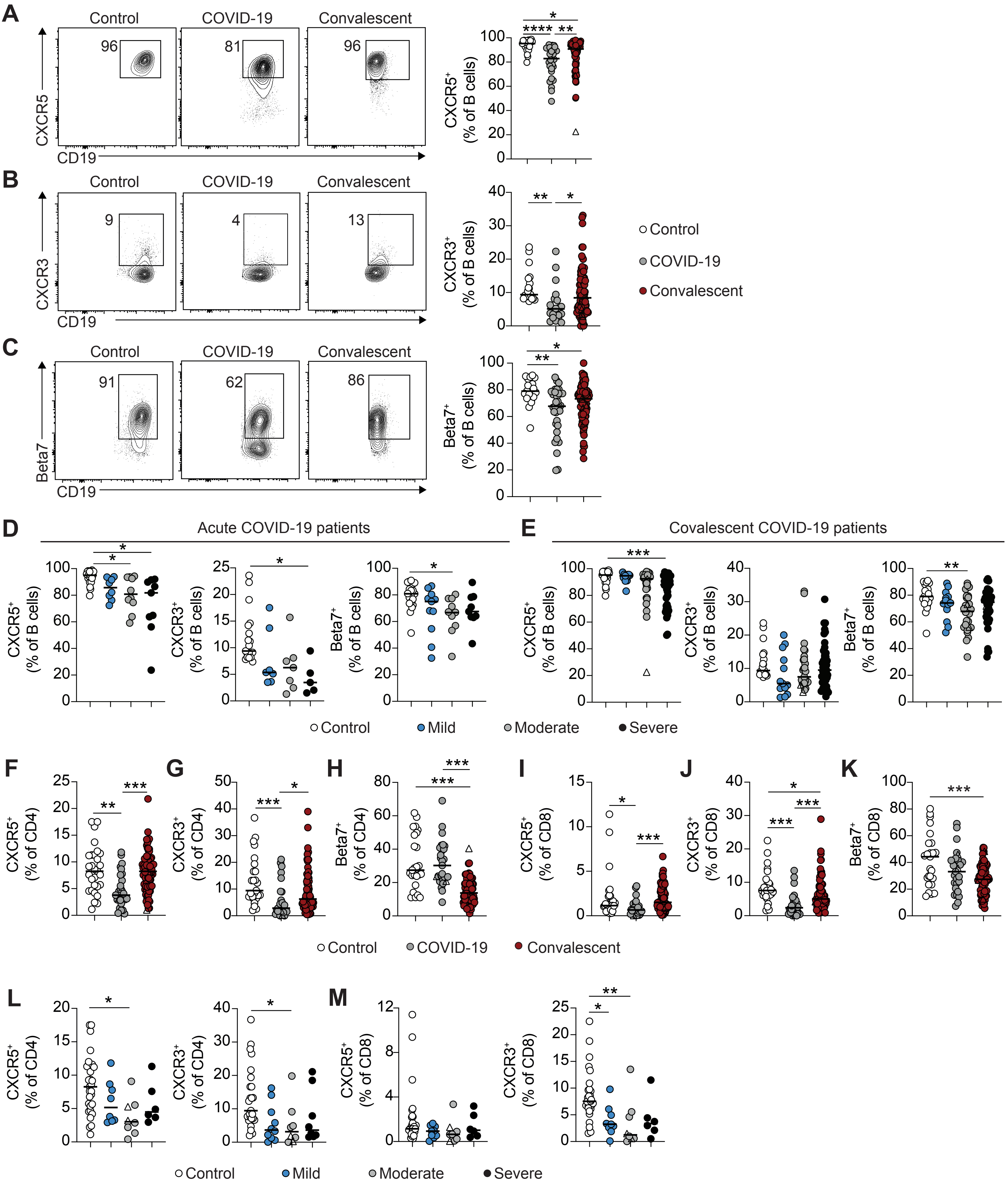

Supplementary Figure 2

Supplemental Figure 2. Altered expression of migratory markers in acute but not convalescent COVID-19 patients, Related to Figures 1 and 2. **(A-C)** Representative flow cytometry plots and graphs showing frequencies of B cells expressing **(A)** CXCR5, **(B)** CXCR3 and **(C)** β7 in healthy individuals (n=19-21), acute (n=21-34) and convalescent (n=81) COVID-19 patients. **(D)** Graphs showing frequencies of B cells expressing CXCR3, CXCR5 and β7 in healthy individuals (n=19) and acute COVID-19 patients with mild (n=7-11), moderate (n=7-10) and severe (n=5-9) disease. **(E)** Graphs showing frequencies of B cells staining positive for CXCR3, CXCR5 and β7 in healthy individuals (n=19-21) and convalescent COVID-19 patients which initially presented with mild (n=13), moderate (n=26) and severe (n=41) disease. **(F-H)** Graphs showing frequencies of $CD4^+$ cells positive for **(F)** CXCR5, **(G)** CXCR3 and **(H)** β7 in healthy individuals (n=25-29), acute (n=26-27) and convalescent (n=80-83) COVID-19 patients. **(I-K)** Graphs showing frequencies of $CD8^+$ cells positive for **(I)** CXCR5, **(J)** CXCR3 and **(K)** β7 in healthy individuals (n=25-29), acute (n=27-28) and convalescent (n=80-81) COVID-19 patients. **(L,M)** Graphs showing frequencies of **(L)** $CD4^+$ and **(M)** $CD8^+$ T cells staining positive for CXCR3 and CXCR5 in healthy individuals (n=25-29) and acute COVID-19 patients with mild (n=8-10), moderate (n=8) and severe (n=6-8) disease. Graphs show individual patient data, with the bar representing median values. In all graphs, triangles represent SARS-CoV-2 PCR-negative patients. *p<0.05, **p<0.01, ***p<0.001, ****p<0.0001, one-way ANOVA with Holm-Sidak post-hoc testing (A-E, K and L (CXCR5)) or Kruskal-Wallis test with Dunn's post-hoc testing (F-J, L(CXCR3), M) for multiple comparisons.

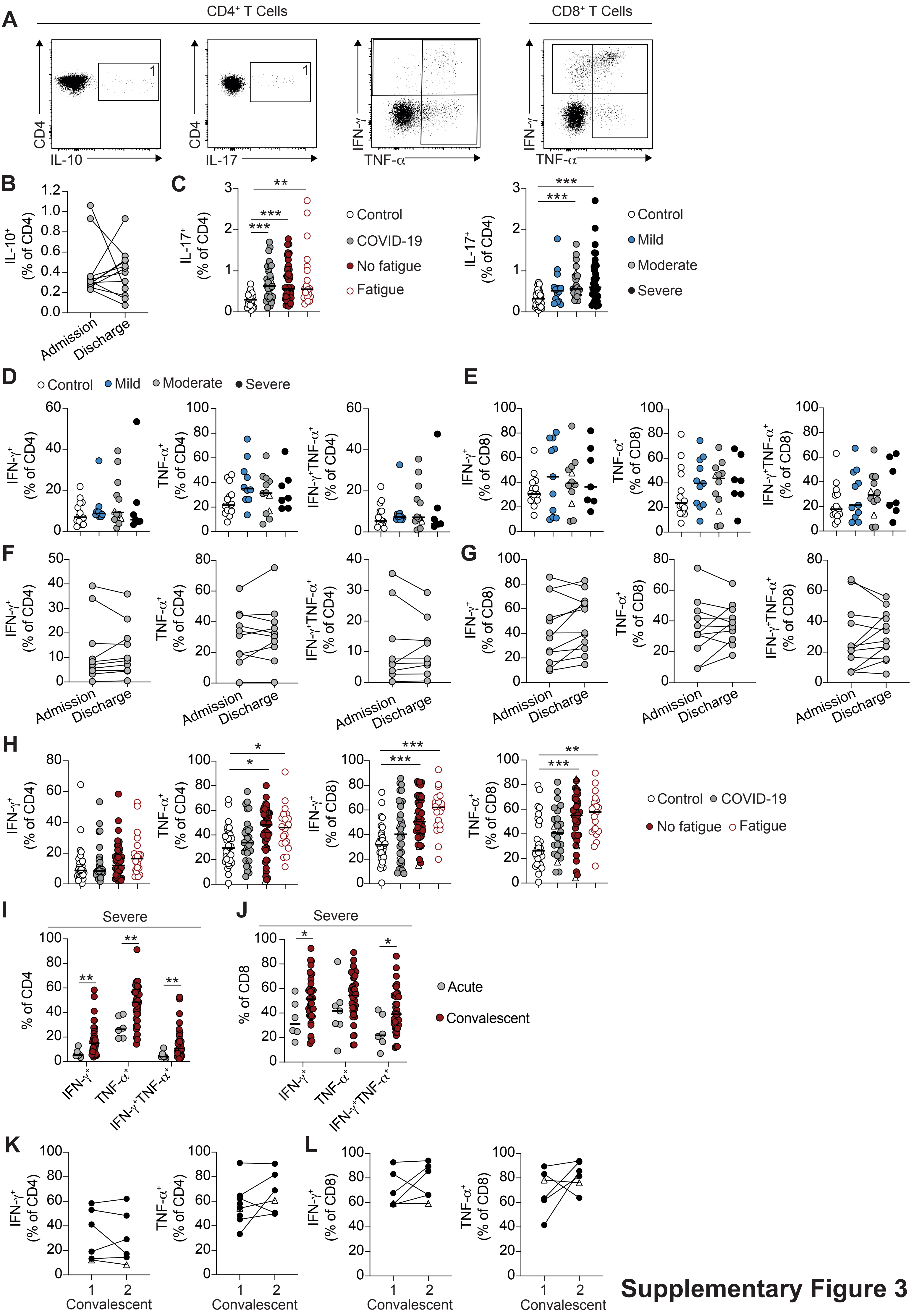

Supplementary Figure 3

Supplemental Figure 3. Long-lasting changes in type-I cytokine production T cells in convalescent COVID-19 patients, Related to Figure 3. **(A)** Representative FACS plots showing example staining for cytokine$^+$ CD4$^+$ and CD8$^+$ T cells. **(B)** Graph shows frequency of IL-10$^+$CD4$^+$ T cells following 3 hour stimulation with PMA and ionomycin in acute COVID-19 patients at the first and last time points of hospitalization. **(C)** Graph show frequencies of IL-17$^+$CD4$^+$ T cells following 3 hour stimulation with PMA and ionomycin in healthy individuals (n=22-30), acute COVID-19 patients (n=31) and convalescent COVID-19 patients stratified by fatigue reporting (not reporting (n=50) and reporting (n=20) enhanced fatigue) and mild (n=14), moderate (n=27) and severe (n=36) acute disease severity. **(D, E)** Graphs show frequencies of (**D**) CD4$^+$ and (**E**) CD8$^+$ T cells which stain positive for IFNγ and TNFα following 3 hour stimulation with PMA and ionomycin in healthy individuals (n=14) and acute COVID-19 patients with mild (n=10-11), moderate (n=12) and severe (n=7) disease. (**F, G**) Graphs show frequencies of **(F)** CD4$^+$ and **(G)** CD8$^+$ T cells that stain positive for IFNγ and TNFα following 3 hour stimulation with PMA and ionomycin in acute COVID-19 patients at the first and last time points of hospitalization. **(H)** Graphs show frequencies of CD4$^+$ and CD8$^+$ T cells which stain positive for IFNγ and TNFα following 3 hour stimulation with PMA and ionomycin in healthy individuals (n=28-30), acute COVID-19 patients (n=24-33) and convalescent COVID-19 patients not reporting (n=44-49) and reporting (n=20-21) enhanced fatigue. **(I, J)** Graphs show frequencies of CD4$^+$ and CD8$^+$ T cells which stain positive for IFNγ and TNFα in all severe COVID-19 patients at acute (grey) and convalescent (maroon) time-points. **(K, L)** Graphs track frequencies of CD4$^+$ and CD8$^+$ T cells which stain positive for IFNγ and TNFα following 3 hour stimulation with PMA and ionomycin in the same COVID-19 patient at convalescent time-points pre (1) and post (2) 6 months since hospital discharge (n=4-6). Graphs show individual patient data, with the bar representing median values. In all graphs, triangles represent SARS-CoV-2 PCR-negative patients. *p<0.05, **p<0.01, ***p<0.001, one-way ANOVA with Kruskal-Wallis test with Dunn's post-hoc testing for multiple comparisons (except for graphs showing CD4$^+$TNFα^+ and CD8$^+$TNFα^+ T cells in D and E where One-way ANOVA with Holm-Sidak post-hoc test was employed).

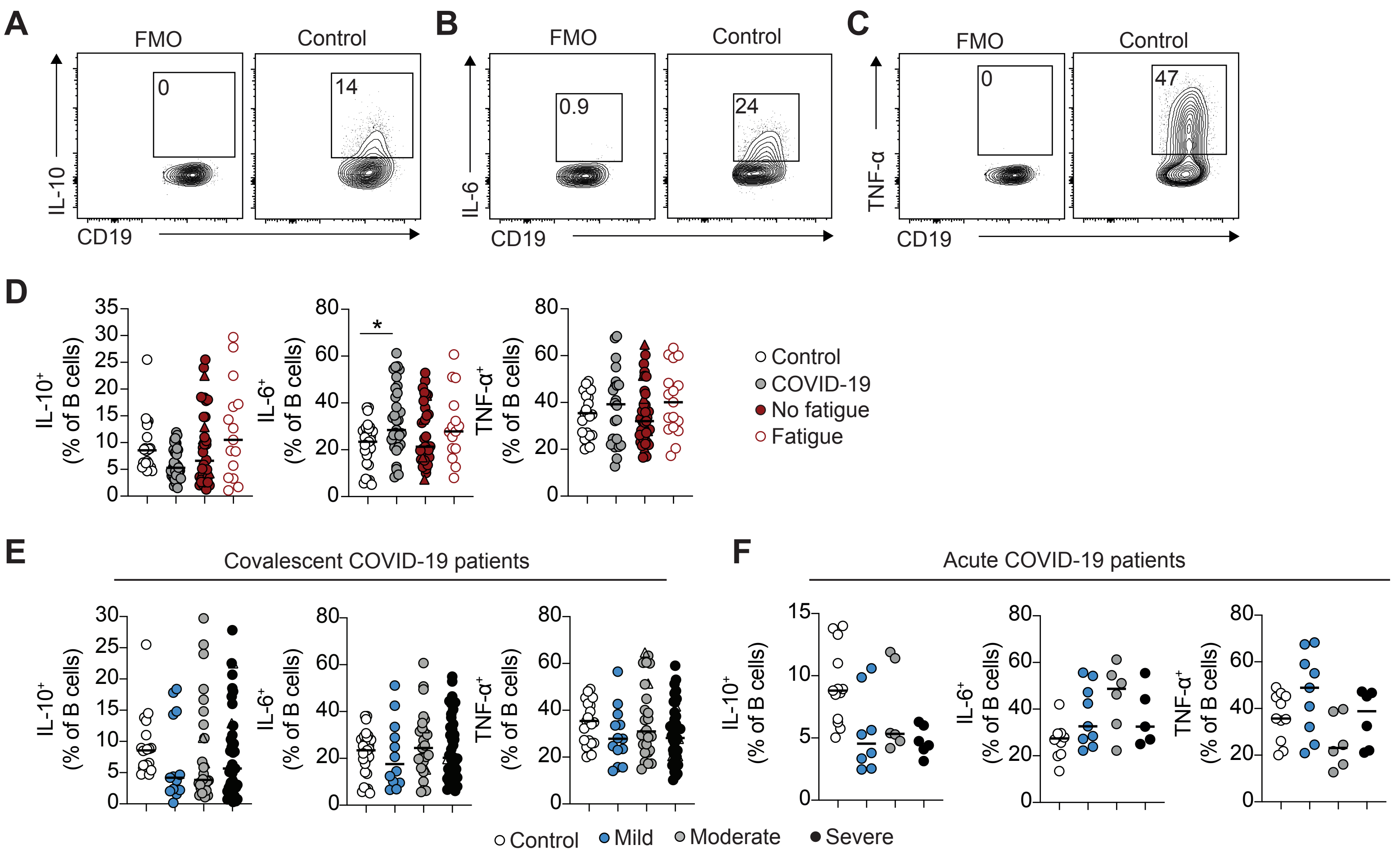

Supplementary Figure 4

Supplemental Figure 4. Cytokine production by B cells from acute and convalescent COVID-19 patients, Related to Figure 3. **(A-C)** Representative flow cytometry plots showing gating strategy of $CD19^+$ B cells positive for IL-10, IL-6 and TNFα following 48 hour stimulation with CpGB. **(D)** Graphs show frequencies of $CD19^+$ B cells staining positive IL-10, IL-6 and TNFα following 48 hour stimulation with CpGC in healthy individuals (n=22-27), acute COVID-19 patients (n=22-37) and convalescent COVID-19 patients not reporting (n=36-38) and reporting (n=15-17) enhanced fatigue. **(E)** Graphs show frequencies $CD19^+$ B cells staining positive for IL-10, IL-6 and TNFα in healthy individuals (n=22-23), and convalescent COVID-19 patients which initially presented with mild (n=12-13), moderate (n=25-26) and severe (n=41) disease. **(F)** Graphs show frequencies $CD19^+$ B cells staining positive for IL-10, IL-6 and TNFα in healthy individuals (n=11-13), and acute COVID-19 patients with mild (n=8-9), moderate (n=6) and severe (n=6) disease. Graphs show individual patient data, with the bar representing median values. *$p<0.05$, one-way ANOVA with Kruskal-Wallis test with Dunn's post-hoc testing for multiple comparisons.
